# M. Rufz on Phthisis

**Published:** 1842-06-18

**Authors:** 


*Phthisis.*—In a report on phthisis made at Martinique by M. Rufz, the reporter observes, that phthisis is less an hereditary disease transmitted from parent to child, than a congenital disorder proper to children issuing from the same family.—*London Lancet.* April 30th, 1842.

